# Supplementary material for: Identification of photoperiod-induced specific miRNAs in the adrenal glands of Sunite sheep (Ovis aries)
Source: Front Vet Sci. 2022 Jul 22;9:888207. doi: 10.3389/fvets.2022.888207 (PMC9354845; doi:10.3389/fvets.2022.888207)
Supplement: Supplementary file 1 [file Data_Sheet_1.ZIP › Supplementary file/miRDeep2 file.zip!/miRDeep2 file/Know_Structure/SP42A3/oar-mir-3959.pdf]

miRBase precursor	: oar-mir-3959
Total read count	: 14645
oar-miR-3959-5p read count	: 13287
oar-miR-3959-3p read count	: 1358
remaining reads	: 0

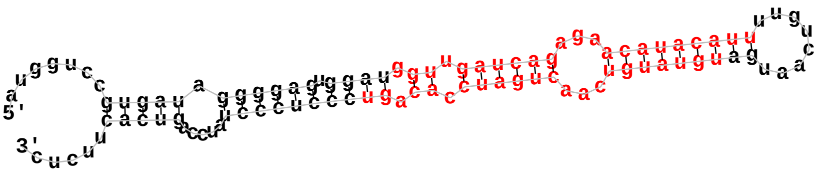

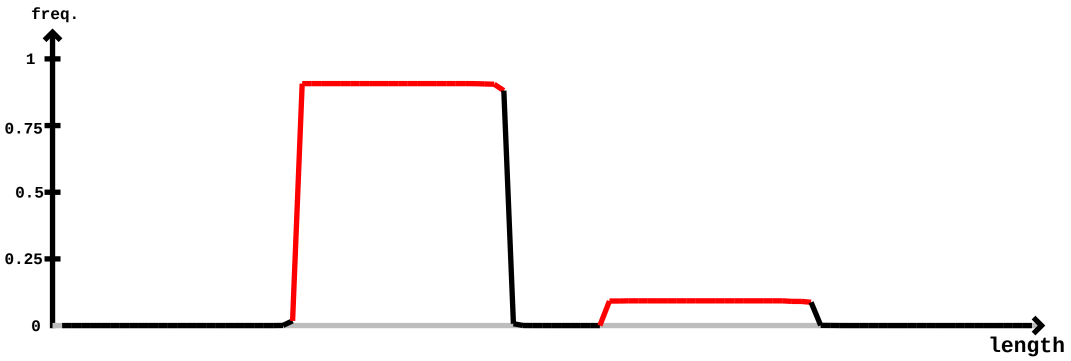


```
                        oar-miR-3959-5p
                                                        oar-miR-3959-3p
5'- augguccgugauaggggaguggaugguugaucagagaacauacauuuugucaaugauguaugucaacugauccacaguccccucccuauccagucacuucuc   -3'    exp
    .......(((((.((((((.((((.((.((((((...(((((((((........)))))))))...)))))).)).))))))))))).....))))).....    reads    mm    sample
    ......................augguugaucagagaacaua...........................................................    1        0     seq
    ......................augguugaucagagaacauac..........................................................    1        0     seq
    ......................augguugaucagagaacauaca.........................................................    4        0     seq
    ......................augguugaucagagaacauacauu.......................................................    1        0     seq
    .......................ugguugaucagagaacauaca.........................................................    2        0     seq
    .......................ugguugaucagagaacauacau........................................................    5        0     seq
    .......................ugguugaucagagaacauacauu.......................................................    242      0     seq
    .......................ugguugaucagagaacauacauuu......................................................    2        0     seq
    ........................gguugaucagagaacaua...........................................................    1        0     seq
    ........................gguugaucagagaacauac..........................................................    15       0     seq
    ........................gguugaucagagaacauaca.........................................................    11       0     seq
    ........................gguugaucagagaacauacau........................................................    344      0     seq
    ........................gguugaucagagaacauacauu.......................................................    12556    0     seq
    ........................gguugaucagagaacauacauuu......................................................    95       0     seq
    ........................gguugaucagagaacauacauuuu.....................................................    3        0     seq
    ........................gguugaucagagaacauacauuuug....................................................    1        0     seq
    .........................guugaucagagaacauacauu.......................................................    3        0     seq
    ........................................................uguaugucaacugaucca...........................    1        0     seq
    ........................................................uguaugucaacugauccac..........................    28       0     seq
    ........................................................uguaugucaacugauccaca.........................    4        0     seq
    ........................................................uguaugucaacugauccacag........................    46       0     seq
    ........................................................uguaugucaacugauccacagu.......................    1266     0     seq
    ........................................................uguaugucaacugauccacaguc......................    2        0     seq
    .........................................................guaugucaacugauccacagu.......................    1        0     seq
    ..........................................................uaugucaacugauccacag........................    1        0     seq
    ..........................................................uaugucaacugauccacagu.......................    1        0     seq
    ..........................................................uaugucaacugauccacagucc.....................    7        0     seq
    ..........................................................uaugucaacugauccacaguccc....................    1        0     seq
```
